# Effect of glycemic control on the risk of pancreatic cancer: A nationwide cohort study

**DOI:** 10.1097/MD.0000000000003921

**Published:** 2016-06-17

**Authors:** Kian-Ching Er, Chen-Yang Hsu, Yi-Kung Lee, Ming-Yuan Huang, Yung-Cheng Su

**Affiliations:** a School of Medicine, Tzu Chi University, Hualien, Taiwan; b Emergency Department, Dalin Tzu Chi Hospital, Buddhist Tzu Chi Medical Foundation, Chiayi, Taiwan; c Department of Public Heath, National Taiwan University, Taipei, Taiwan; d Department of Emergency Medicine, Mackay Memorial Hospital, Taipei, Taiwan.

**Keywords:** diabetes, glycemic control, pancreatic cancer

## Abstract

Although the relationship between diabetes and pancreatic cancer has been studied, the effects of glycemic control on pancreatic cancer have never been evaluated. This study investigates the relationship between glycemic control and pancreatic cancer.

Data from 1 million National Health Insurance beneficiaries were screened. The study cohort consisted of 46,973 diabetic patients and 652,142 nondiabetic subjects. Of the patients with diabetes, 1114 who had been admitted for hyperglycemic crisis episodes were defined as having poorly controlled diabetes. All adult beneficiaries were followed from January 1, 2005 to December 31, 2013, to determine whether pancreatic cancer was diagnosed. The Cox regression model was applied to compare the adjusted hazards for potential confounders.

After controlling for age, sex, urbanization level, socioeconomic status, chronic liver disease, hypertension, coronary artery disease, hyperlipidemia, malignancies, smoking, chronic obstructive pulmonary disease, obesity, history of alcohol intoxication, chronic renal insufficiency, biliary tract disease, chronic pancreatitis, Charlson Comorbidity Index score, and high-dimensional propensity score, the adjusted hazard ratio of pancreatic cancer was 2.53 (95% confidence interval 1.96–3.26) in patients with diabetes. In diabetic patients with poor glycemic control, the hazard ratio of pancreatic cancer was significantly higher (hazard ratio 3.61, 95% confidence interval 1.34–9.78).

This cohort study reveals a possible relationship between diabetes and pancreatic cancer. Moreover, poorly controlled diabetes may be associated with a higher possibility of pancreatic cancer.

## Abstract 2

Supplemental Digital Content is available in the text

## 1 Introduction

Pancreatic cancer is a highly lethal malignancy. Despite improvements in therapeutics for most other cancers in recent years, the mortality rate for pancreatic cancer remains high, with a 5-year survival rate of about 6%.^[1,2]^ Because symptoms are initially absent, diagnosis of the disease is often delayed until later stages, making treatment more difficult.^[3,4]^ Early identification of patients at risk for pancreatic cancer might promote earlier detection of this disease. Several risk factors for pancreatic cancer, including smoking, obesity, chronic pancreatitis, and family history, have been evaluated.^[1,5]^

Recently, increasing epidemiological evidence indicates an association between diabetes and pancreatic malignancy.^[6–8]^ Although the mechanism of this association is still unclear, emerging molecular studies suggest that the tumorigenic effect of hyperglycemia, the mitogenic effect of obesity-associated hyperinsulinemia, and the chronic inflammation in diabetes might be involved in the proliferation and metastasis of pancreatic cancer.^[8–10]^ However, few studies have evaluated the relationship between glycemic control and pancreatic cancer.

The present study used a large institutional database to investigate the possible risk of pancreatic cancer in diabetic patients in Taiwan. In the previous study, we noted that diabetic patients with poor compliance may have higher risk of acute pancreatitis compared with the general diabetes population.^[11]^ We hypothesized that diabetes is associated with an increased risk of pancreatic cancer and that those diabetic patients with poor glycemic control may have a higher possibility of pancreatic cancer. High-dimensional propensity score (hdPS) analysis (a semiautomated statistical method) was used to address possible unmeasured confounding factors. Given the increasing incidence of diabetes around the world, the results of this study may address more awareness about the association among glycemic control and pancreatic cancer.

## 2 Methods

### 2.1 Ethics statement

This study was conducted after approval from the Institutional Review Board of Dalin Tzu Chi Hospital, Buddhist Tzu Chi Medical Foundation, Taiwan. Since all information indicating patient identity was removed before analysis, the review board waived the requirement for written informed consent from the included patients.

### 2.2 Database

The Taiwanese National Health Insurance (NHI) program was introduced in 1995.^[12]^ The Longitudinal Health Insurance Database 2005—a dataset comprising records from 1 million individuals—was randomly chosen as a representative cohort from a larger database including all beneficiaries of the NHI program. Statistically significant differences were not observed for this group as compared with the larger cohort with respect to age, sex, or healthcare costs, according to the Taiwan National Health Research Institute.^[13]^

### 2.3 Study population

The representative cohort of 1 million individuals was tracked between January 1, 2003 and December 31, 2013. All individuals over 18 years of age who were living in 2005 were initially identified. The ambulatory care claim records of those diagnosed with diabetes [International Classification of Diseases, Ninth Revision, Clinical Modification (ICD-9-CM) code: 250] were then examined for data entered during the follow-up period. To avoid misclassification, an individual could be classified as having diabetes only if he or she was diagnosed with diabetes and then experienced another 1 or more diagnoses within the subsequent 12 months. In addition, the time between the first and last visits during the follow-up period had to be more than 30 days to avoid accidental inclusion of patients with miscoded diagnoses.^[14]^ Poorly controlled diabetes was defined as a hospitalization with the diagnosis of a hyperglycemic crisis episode (HCE) (ICD-9-CM codes: diabetic ketoacidosis, 250.1, or hyperosmolar hyperglycemic state, 250.2).^[11,15,16]^ These selection processes and definitions were well-validated with high positive predictive values in previous studies.^[14,17]^

To avoid financial burden for patients with major illnesses, the NHI specifies 31 categories of catastrophic illness (e.g., malignancies, major depression, and chronic renal failure) for which no copayment is charged, once reviewed and approved by a committee. To maximize case accuracy, only patients registered with pancreatic cancer for the catastrophic illness certificate (ICD-9-CM code: 157) were enrolled.

**Figure 1:**
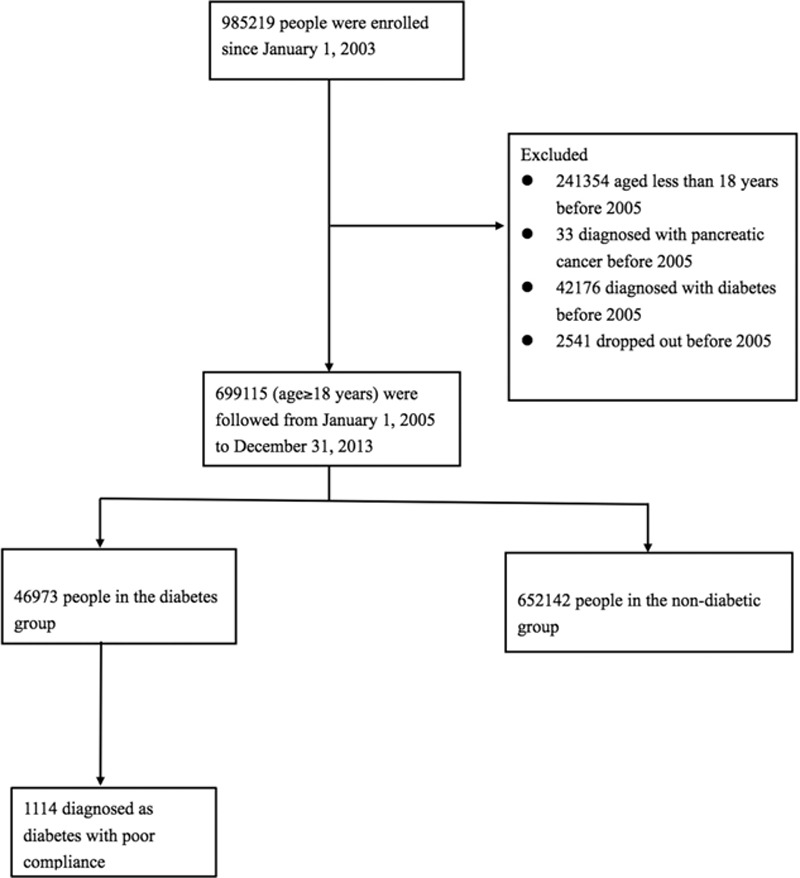
Flow diagram of the population-based study.

After excluding patients with diabetes and pancreatic cancer before January 1, 2005, 46,973 patients were included in the diabetic group and 652,142 in the nondiabetic group. For each diabetic patient, the date of his or her first diagnosis was considered the index date. The index date for subjects in the nondiabetic group was set as January 1, 2005. Subjects in the diabetic and nondiabetic groups were then followed through December 31, 2013 for possible diagnosis of pancreatic cancer. Cases were censored for patients who were no longer beneficiaries of the NHI Program (i.e., death or transfer out) or who were still robust at the end of the follow-up period (Fig. 1).

### 2.4 Prespecified covariates

To better characterize the relationship between diabetes and pancreatic cancer, several covariates were used, including age, sex, urbanization level, and socioeconomic status (SES). The age of each patient was defined as the difference between the index date and the date of birth. Income-related insurance payment amounts were used as a proxy measure of individual SES at follow-up.

Additionally, specific comorbid conditions reported to be associated with pancreatic cancer (chronic liver disease, hypertension, coronary artery disease, hyperlipidemia, malignancies, smoking, chronic obstructive pulmonary disease, obesity, history of alcohol intoxication, chronic renal insufficiency, biliary tract disease, and chronic pancreatitis)^[8,18–22]^ and the Charlson Comorbidity Index (CCI) score were selected according to discharge diagnoses after either outpatient clinic visits or hospitalizations before January 1, 2005. The detailed ICD-9-CM codes for comorbidities are described elsewhere, and the processes used for selecting comorbidities are standard and widely accepted.^[17,23,24]^

### 2.5 High-dimensional propensity scores

The hdPS is a multistep, empirically-driven algorithm that is used to adjust for confounding factors.^[17,25]^ It automatically assesses code repetition, prioritizes covariates, and identifies covariates for adjustment. In this study, candidate covariates from predefined data dimensions including clinical procedures received, medications administered based on the Anatomical Therapeutic Chemical (ATC) Classification System, and ICD-9-CM code diagnoses reported from January 1, 2003 to December 31, 2004, were identified using the SAS macro. We selected 1000 variables most likely to result in a bias for adjustments. These variables were analyzed with a logistic regression model to generate the predicted probability (propensity score [PS]) of diabetes when compared with those of the nondiabetic population. The PS was then added into analyses as a summary variable.

### 2.6 Statistical analysis

Age and PS were taken as continuous variables; all other covariates were considered as categorical variables. Categorical and continuous variables were compared using Pearson chi-square test and a *t* test, respectively, to evaluate baseline heterogeneity. The cumulative risks of pancreatic cancer were first determined by plotting Nelson–Aalen curves. The hazard ratios (HRs) for pancreatic cancer in patients with diabetes were calculated using the Cox proportional-hazard regression model after adjustment for age, sex, urbanization level, SES, chronic liver disease, hypertension, coronary artery disease, hyperlipidemia, malignancies, smoking, chronic obstructive pulmonary disease, obesity, history of alcohol intoxication, chronic renal insufficiency, biliary tract disease, chronic pancreatitis, CCI, and PS.

Further analysis was performed to evaluate the association of pancreatic cancer in diabetic patients with poor control to determine whether the relationship between diabetes and pancreatic cancer was dependent on glycemic control. The same analyses were carried out on these subgroups. The SAS statistical package version 9.4 (SAS Institute, Inc., Cary, NC) and STATA version 11.2 (StataCorp, College Station, TX) were used for data analysis. Two-tailed *P* values <0.05 were considered significant.

## 3 Results

**Figure 2:**
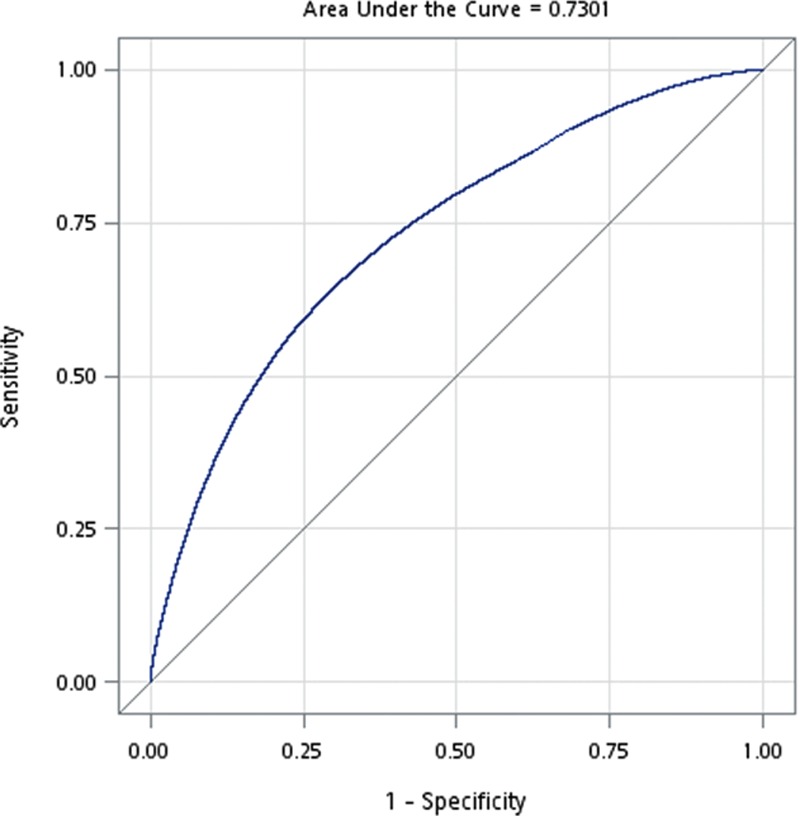
AUC of PS in the prediction of diabetes showing good accuracy. AUC = area under the curve, PS = propensity score.

The distribution of demographic characteristics and selected comorbidities are summarized in Table 1. There were 46,973 patients in the diabetes group and 652,142 in the nondiabetic group. The total follow-up times were 207,861 and 5,568,462 person-years, and the average follow-up period was 4.3 and 8.5 years, respectively. Patients with diabetes were predominantly male and significantly older. They were also more likely to have lower SES, rural area residence, a higher CCI score, chronic liver disease, hypertension, coronary artery disease, hyperlipidemia, malignancies, chronic obstructive pulmonary disease, obesity, history of alcohol use, chronic renal insufficiency, biliary tract disease, chronic pancreatitis, and higher PS. The area under the curve (AUC) of PS in the prediction of diabetes is 0.73, indicating good accuracy (Fig. 2).

**Table 1:**
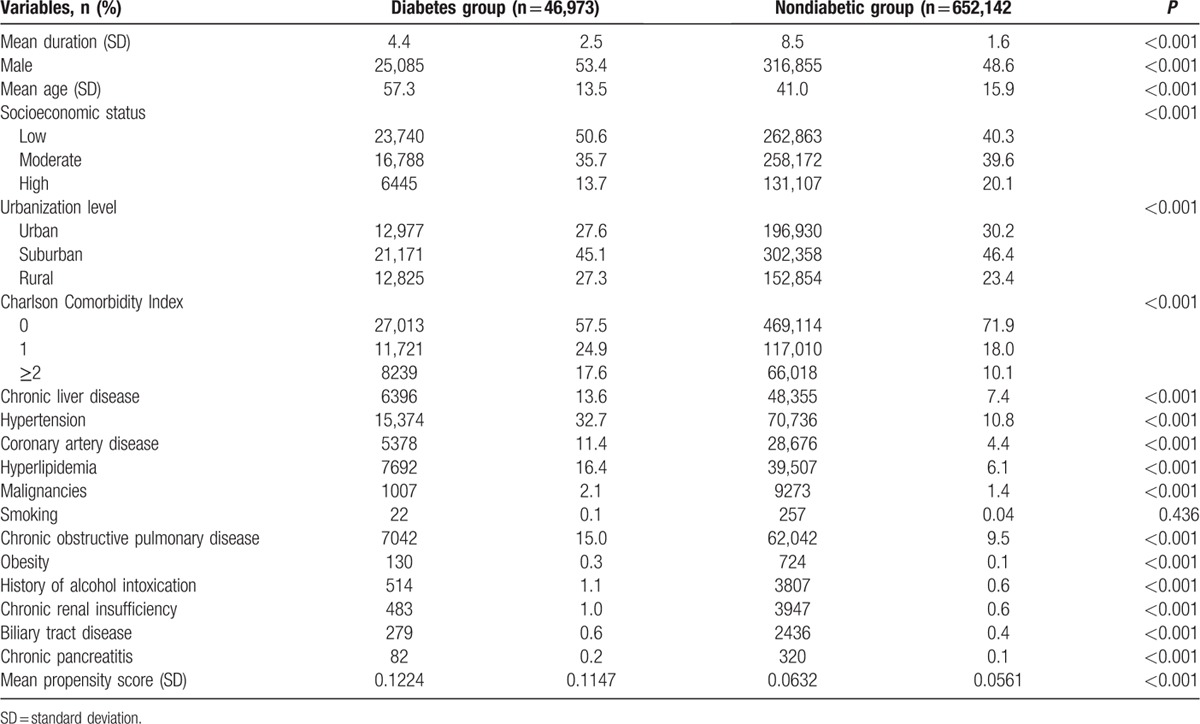
Baseline characteristics of the diabetes group and the non-diabetic group.

**Figure 3:**
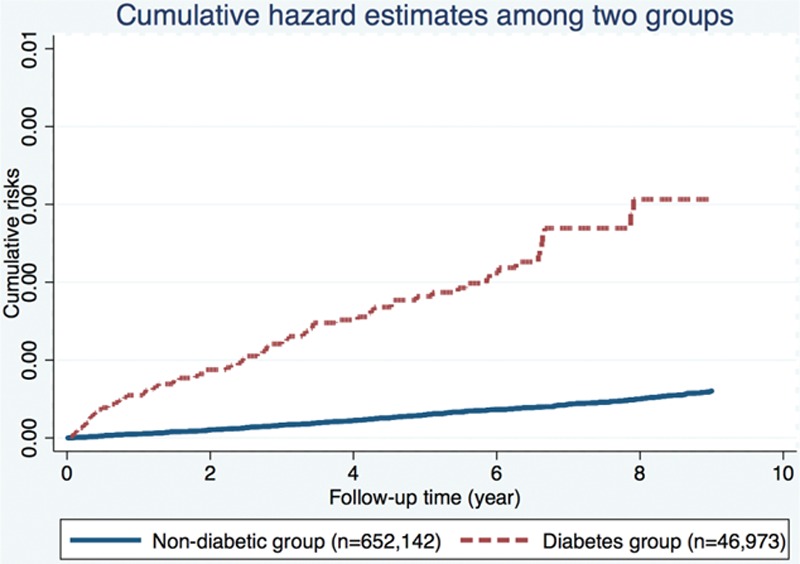
Nelson–Aalen curves showing a higher cumulative risk of pancreatic cancer in the diabetes group.

At the end of follow-up, 497 patients (82 with diabetes, 415 without) had a diagnosis of pancreatic cancer. The crude HR of pancreatic cancer in diabetes patients compared with the general population was 6.12 (95% confidence interval [CI] 4.79–7.82). The Nelson–Aalen plot also showed higher cumulative risk of pancreatic cancer in the diabetes groups (Fig. 3).

A multivariate Cox regression model was then applied to determine the adjusted HRs for pancreatic cancer after controlling for the above-mentioned covariates. A higher HR was still observed for diabetic patients (2.53; 95% CI 1.96–3.26). Other independent risk factors for pancreatic cancer included male sex, older age, lower SES, malignancy, biliary tract disease, chronic pancreatitis, and PS. Findings with relevant statistics are summarized in Table 2. We also managed to achieve the comparability of the study groups by performing a matching technique on age and sex. With a nondiabetes-to-diabetes ratio of 4, 183,672 nondiabetic patients and 45,918 diabetic patients were selected. (Appendix Table 1) The statistical results were similar and summarized in Appendix Table 2.

**Table 2:**
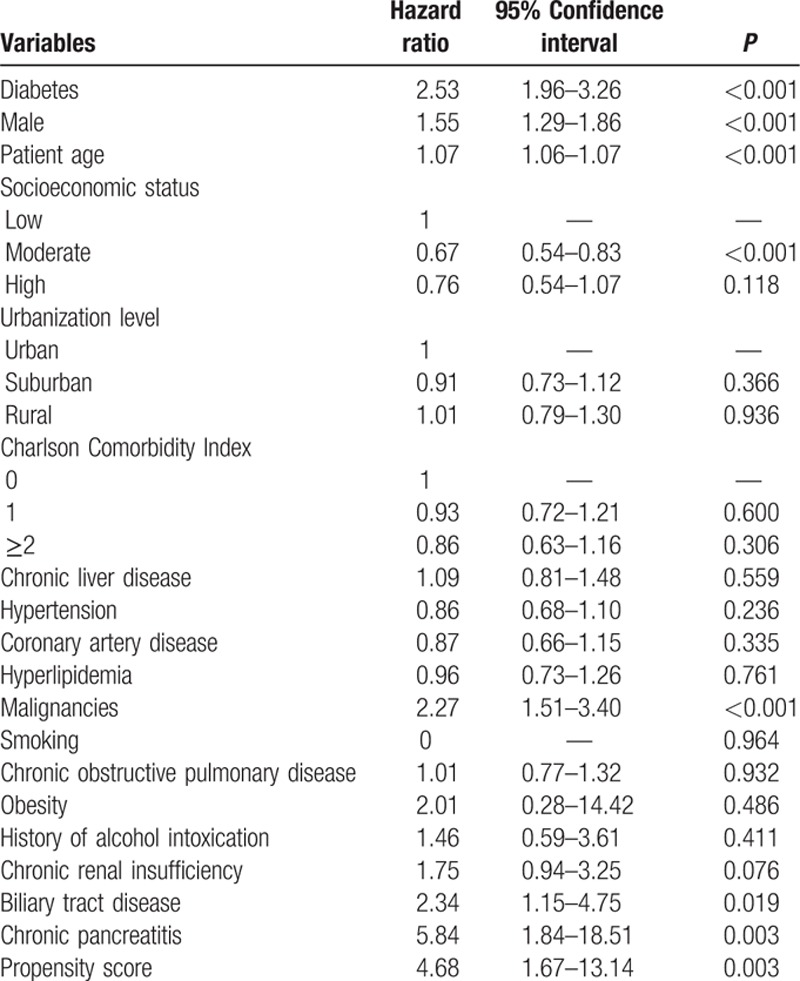
Adjusted hazard ratios of pancreatic cancer for patients with diabetes.

For the 1114 patients further defined as poorly controlled diabetes, the distribution of demographic characteristics and selected comorbidities is shown in Table 3. The total follow-up time were 5532 person-years, and the average follow-up period was 5.0 years. In this subgroup, 4 patients were diagnosed with pancreatic cancer. A multivariate Cox regression model including the same covariates was applied to the restricted subpopulation of poorly controlled diabetes mellitus to assess the effect of hyperglycemic status on incident pancreatic cancer. The adjusted HR for poorly controlled diabetes group compared with nondiabetes group was estimated as 3.61 (95% CI 1.34–9.78) (Table 4). The trend test for the hyperglycemic states of diabetes mellitus and poorly controlled diabetes mellitus adjusting for relevant factors showed a significant result (*P* < 0.001).

**Table 3:**
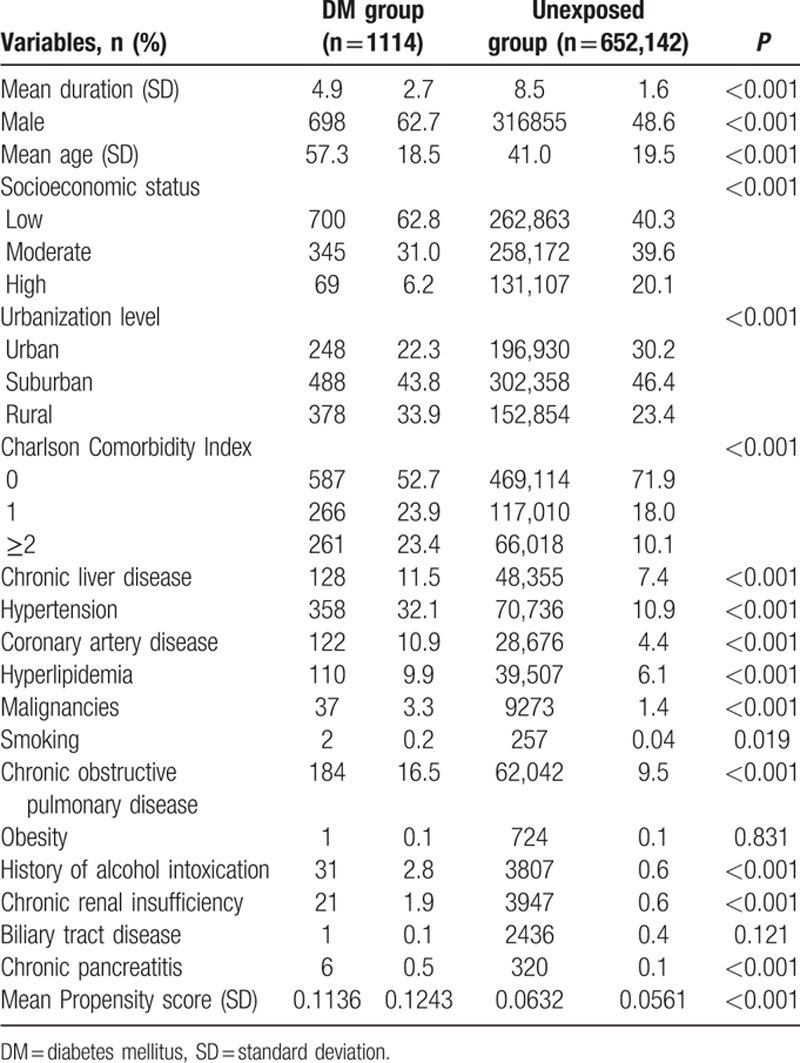
Baseline characteristics of the diabetic patients with poor control.

**Table 4:**
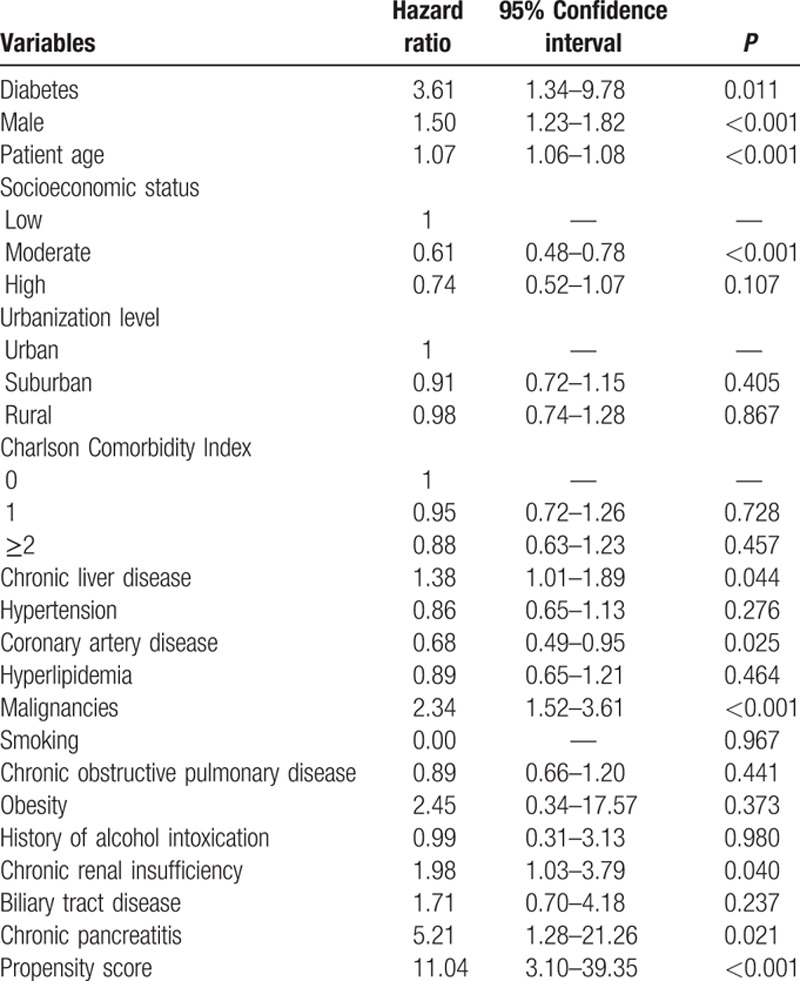
Adjusted hazard ratios of pancreatic cancer for diabetic patients with poor control.

## 4 Discussion

We observed that the risk of pancreatic cancer is significantly higher in patients with diabetes (HR 2.53) than in nondiabetic subjects, and these results are compatible with previous studies.^[8,19,21,26]^ We also found that diabetic patients with a history of HCE had a higher HR (3.61) than did those without HCE, suggesting a possible “severity–response” effect between diabetes and the risk of pancreatic cancer that has not been previously recognized. To our knowledge, this is the first study to address the effect of glycemic control on the relationship between diabetes and pancreatic cancer. The database used in this study is representative of the whole population in Taiwan; as a result, losses to follow-up or selection bias are not concerns.

The mechanism whereby diabetes increases the risk of pancreatic cancer remains unknown, but several hypotheses have been presented. Hyperglycemia is associated with increased reactive oxygen species production and oxidative stress, which may be involved in the pathogenesis and invasiveness of pancreatic cancer.^[8–10,27]^ Insulin resistance-related hyperinsulinemia might increase cancer progression by increasing cell proliferation, decreasing apoptosis, and enhancing cell responsiveness to other growth factors such as insulin-like growth factor.^[19,28,29]^ Diabetes might alter the regulation of hormones such as adiponectin and leptin, which in turn leads to cancer growth via multiple signaling pathways.^[19,30]^ Although further studies are required to investigate the biological mechanism of pancreatic cancer development in diabetic patients, a reasonable and intuitive strategy to further decrease this risk is to achieve better glycemic control.

One strength of our study is that we extensively adjusted for possible unmeasured confounding factors by applying hdPS analysis. In our study, as many as 1000 covariates were generated for analysis. Using an institutional database, possible risk factors for pancreatic cancer (such as family history, unreported smoking, or alcohol consumption status) cannot be assessed. However, a group of covariates might indirectly represent the overall status of patients and further constitute a good overall proxy for unmeasured confounding factors.^[31]^ The use of a large number of proxy covariates for PS estimation may improve the control of confounding.^[25]^

### 4.1 Limitations

Our study has several limitations. First, our findings were derived from institutional data. The exposures and outcomes were recorded using ICD-9-CM diagnosis codes, and laboratory data such as glucose levels and hemoglobin A1c were not available in this database. As an alternative, we defined diabetic patients with poor control as those who had experienced at least 1 episode of HCE, since uncontrolled diabetes is the most common precipitating factor of HCE.^[15]^ Although HCE is an extremely severe state that may not be identical to “poor control,” if the definition failed to differentiate these patients from average diabetic patients, a higher HR would not have been observed.

Second, because of the limitation of the institutional database, we believe the ICD-9-CM diagnostic codes did not perfectly differentiate type I diabetes from type II. As a result, we decided not to perform subgroup analysis of the association between different types of diabetes or HCE and pancreatic cancer. Individualized studies are better options to solve the study question.

Third, we intended to elucidate the association between the glycemic exposure and further development of pancreatic cancer by applying the analysis restricted to subjects with poorly controlled diabetes. However, the restriction results in decreased sample size of event (pancreatic cancer). The observed subjects with incident case of pancreatic cancer among the 3 study groups of nondiabetes, diabetes, and poorly controlled diabetes were 415, 78, and 4, respectively, leading to a wide CI.

Fourth, medications are considered as a possible factor which may alter the incidence of pancreatic cancer in diabetic patients. For example, studies have shown a survival benefit in diabetic patients with pancreatic cancer that have been treated with metformin compared with patients treated with insulin or sulfonylureas.^[32,33]^ However, there is limitation on applying registry data to elucidate the association between medication history and the occurrence of pancreatic cancer. Further study should be conducted to specifically deal with this interesting topic.

Fifth, although the relationships between diabetes and pancreatic cancer are obvious in our results, based on the nature of cohort study, we could not confirm that diabetes is either a true risk factor of pancreatic cancer or merely an association. Further studies focused on the biologic mechanisms should be conducted. Finally, bidirectional relationships between diabetes and pancreatic cancer have been reported,^[34]^ and we acknowledge that reverse causation (i.e., undiagnosed pancreatic cancer resulted in diabetes) is a possibility. However, reverse causation could not fully explain the “severity–response” effect observed in this study. Finally, although we extensively adjusted for possible comorbidities, unmeasured cofounding is still an issue. Based on the nature of our dataset, we could not adjust for some important risk factors such as body mass index, diet, and family history. However, the HRs are significant enough that residual confounding may not fully explain the results. Furthermore, the “severity–response” effect found in the association between diabetes and pancreatic cancer cannot be explained by confounding.

## 5 Conclusions

This study reveals a possible relationship between diabetes and pancreatic cancer. Moreover, poorly controlled diabetes may be associated with a much higher possibility of pancreatic cancer. This finding highlights the importance and benefits of glycemic control in diabetic patients.

## Supplementary Material

Supplemental Digital Content
