# Supplementary material for: Effect of glycemic control on the risk of pancreatic cancer: A nationwide cohort study
Source: Medicine (Baltimore). 2016 Jun 17;95(24):e3921. doi: 10.1097/MD.0000000000003921 (PMC4998487; doi:10.1097/MD.0000000000003921)
Supplement: Supplemental Digital Content [file medi-95-e3921-s001.doc]

| **Appendix Table 1. Baseline characteristics of the matched cohort** | | | | | |  |
| --- | --- | --- | --- | --- | --- | --- |
|  |  | |  |  | |  |
| **Variables, n(%)** | **Diabetes group**  **n=45918** | |  | **Non-diabetic group**  **n=183672** | | ***P-*value** |
| **Mean Duration (SD)** | 4.4 | 2.5 |  | 8.3 | 1.9 | **<0.001** |
| **Male** | 24503 | 53.4 |  | 98257 | 53.5 | 0.608 |
| **Mean age (SD)** | 57.2 | 13.6 |  | 57.2 | 13.6 | 0.451 |
| **Event** | 81 | 0.2 |  | 276 | 0.2 | 0.204 |
| **Socioeconomic status** |  |  |  |  |  | **<0.001** |
| Low | 23188 | 50.5 |  | 99465 | 54.2 |  |
| Moderate | 16426 | 35.8 |  | 62123 | 33.8 |  |
| High | 6304 | 13.7 |  | 22084 | 12.0 |  |
| **Urbanization level** |  |  |  |  |  | **<0.001** |
| Urban | 12696 | 27.6 |  | 50332 | 27.4 |  |
| Suburban | 20704 | 45.1 |  | 79997 | 43.6 |  |
| Rural | 12518 | 27.3 |  | 53343 | 29.0 |  |
| **Charlson Comorbidity Index** |  |  |  |  |  | **<0.001** |
| 0 | 26458 | 57.6 |  | 104628 | 57.0 |  |
| 1 | 11430 | 24.9 |  | 43894 | 23.9 |  |
| ≥ 2 | 8030 | 17.5 |  | 35150 | 19.1 |  |
| **Chronic liver disease** | 6225 | 13.6 |  | 19313 | 10.5 | **<0.001** |
| **Hypertension** | 14966 | 32.6 |  | 47830 | 26.0 | **<0.001** |
| **Coronary artery disease** | 5230 | 11.4 |  | 19510 | 10.6 | **<0.001** |
| **Hyperlipidemia** | 7492 | 16.3 |  | 22162 | 12.1 | **<0.001** |
| **Malignancies** | 971 | 2.1 |  | 5645 | 3.1 | **<0.001** |
| **Smoking** | 21 | 0.1 |  | 99 | 0.1 | 0.493 |
| **Chronic obstructive pulmonary disease** | 6875 | 15.0 |  | 28486 | 15.5 | **0.004** |
| **Obesity** | 126 | 0.3 |  | 157 | 0.1 | **<0.001** |
| **History of alcohol intoxication** | 496 | 1.1 |  | 1322 | 0.7 | **<0.001** |
| **Chronic renal insufficiency** | 467 | 1.0 |  | 2480 | 1.4 | **<0.001** |
| **Biliary tract disease** | 275 | 0.6 |  | 1177 | 0.6 | 0.311 |
| **Chronic pancreatitis** | 81 | 0.2 |  | 125 | 0.1 | **<0.001** |
|  |  |  |  |  |  |  |

| **Appendix Table 2. Adjusted HRs of pancreatic cancer for patients with diabetes** | | | | |
| --- | --- | --- | --- | --- |
|  |  |  | |  |
| **Variables** |  | **Hazard Ratio 95% confidence interval** | | ***P-*value** |
| **Diabetes** |  | 2.50 | 1.94-3.22 | **<0.001** |
| **Socioeconomic status** |  |  |  |  |
| Low |  | 1 | -- | -- |
| Moderate |  | 0.58 | 0.45-0.74 | **<0.001** |
| High |  | 0.43 | 0.28-0.66 | **<0.001** |
| **Urbanization level** |  |  |  |  |
| Urban |  | 1 | -- | -- |
| Suburban |  | 0.96 | 0.74-1.24 | 0.744 |
| Rural |  | 1.31 | 0.98-1.74 | 0.065 |
| **Charlson Comorbidity Index** |  |  |  |  |
| 0 |  | 1 | -- | -- |
| 1 |  | 1.06 | 0.78-1.44 | 0.715 |
| ≥ 2 |  | 1.01 | 0.72-1.42 | 0.952 |
| **Chronic liver disease** |  | 0.68 | 0.45-1.02 | 0.060 |
| **Hypertension** |  | 1.43 | 1.11-1.83 | **0.005** |
| **Coronary artery disease** |  | 1.10 | 0.79-1.53 | 0.561 |
| **Hyperlipidemia** |  | 1.08 | 0.80-1.45 | 0.634 |
| **Malignancies** |  | 2.38 | 1.46-3.88 | **0.001** |
| **Smoking** |  | 0 | 0-0 | **<0.001** |
| **Chronic obstructive pulmonary disease** |  | 1.32 | 0.98-1.78 | 0.065 |
| **Obesity** |  | 2.06 | 0.29-14.63 | 0.471 |
| **History of alcohol intoxication** |  | 1.63 | 0.60-4.46 | 0.340 |
| **Chronic renal insufficiency** |  | 1.81 | 0.88-3.72 | 0.109 |
| **Biliary tract disease** |  | 3.31 | 1.57-6.99 | **0.002** |
| **Chronic pancreatitis** |  | 5.43 | 1.43-20.58 | 0.013 |
|  |  |  |  |  |
